# Retraction: HIV/AIDS in Indonesia: current treatment landscape, future therapeutic horizons, and herbal approaches

**DOI:** 10.3389/fpubh.2026.1884453

**Published:** 2026-05-26

**Authors:** Frontiers Editorial Office

The journal retracts the 14 February 2024 article cited above.

Following publication, concerns were raised regarding the scientific validity of this article and the potential undeclared use of generative AI. It was determined that the complaints were valid and that the article does not meet the standards of editorial and scientific soundness for Frontiers in Public Health. During our investigation, the authors confirmed that generative AI was used during manuscript preparation, which was not declared at the time of submission.

Given the extent of the concerns regarding the reliability and soundness of the article, and the authors' acknowledgment of undeclared generative AI use, Frontiers no longer has confidence in the article and has therefore retracted it.

The authors agree to this retraction.

Frontiers would like to thank Alex Glynn for contacting the journal regarding the published article.

